# The dangers of damage control orthopedics: a case report of vascular injury after femoral fracture external fixation

**DOI:** 10.1186/1754-9493-6-7

**Published:** 2012-03-26

**Authors:** Gregory R Staeheli, Michael R Fraser, Steven J Morgan

**Affiliations:** 1 Department of Orthopaedic Surgery, Naval Medical Center San Diego, San Diego, CA, USA; 2 Department of Orthopaedic Surgery, Swedish Medical Center, Englewood, CO, USA; 3 Denver Health Hospital, Denver, CO, USA; 4 Naval Medical Center San Diego, 34800 Bob Wilson Drive, Ste 112, San Diego, CA 92134, USA

**Keywords:** Artery occlusion, Vascular injury, External fixator, Open fracture, Femur

## Abstract

**Background:**

Placement of external fixation frames is an expedient and minimally invasive method of achieving bone and joint stability in the setting of severe trauma. Although anatomic safe zones are established for placement of external fixation pins, neurovascular structures may be at risk in the setting of severe trauma.

**Case report:**

We present a case of a 21-year-old female involved in a high speed motorcycle accident who sustained a Type IIIB open segmental femur fracture with significant thigh soft tissue injury. Damage control orthopedic principals were applied and a spanning external fixator placed for provisional femoral stabilization. Intraoperative vascular examination noted absent distal pulses, however an intraoperative angiogram showed arterial flow distal to the trifurcation. Immediately postoperatively the dorsalis pedis pulse was detected using Doppler ultrasound but was then non-detectable over the preceding 12-hours. Femoral artery CT angiogram revealed iatrogenic superficial femoral artery occlusion due to kinking of the artery around an external fixator pin. Although the pin causing occlusion was placed under direct visualization, the degree of soft tissue injury altered the appearance of the local anatomy. The pin was subsequently revised allowing the artery to travel in its anatomic position, restoring perfusion.

**Conclusion:**

This case highlights the dangers associated with damage control orthopedics, especially when severe trauma alters normal local anatomy. Careful assessment of external fixator pin placement is crucial to avoiding iatrogenic injury. We recommend a thorough vascular examination pre-operatively and prior to leaving the operating room, which allows any abnormalities to be further evaluated while the patient remains in a controlled environment. When an unrecognized iatrogenic injury occurs, serial postoperative neurovascular examinations allow early recognition and corrective actions.

## Background

Damage control orthopedics (DCO) is a principal that immediate definitive fixation of long-bone fractures can be detrimental to severely injured patients who are physiologically unstable [1]. However, the application of this concept in terms of method of interim stabilization and timing of definitive fixation remains a topic of debate. Placement of external fixation frames is an expedient and minimally invasive method of achieving bone and joint stability in the setting of severe trauma and ipsilateral vascular injury [2,3]. If applied safely, the use of these frames as a temporizing measure has been shown to be beneficial in preventing the second-hit inflammatory phenomenon and is associated with decreased blood loss, shorter operative time, low DVT rates, and decreased mortality in the severely traumatized patient [2-7]. Early conversion (< 2 weeks) of temporary external fixation frames to intramedullary nails has shown to produce high union rates and comparable infection rates to primary intramedullary nailing [3,8]. Definitive external fixation, however, is associated with a high rate of complications which include pin site infection, nonunion, knee stiffness, and neurovascular injury [9].

External fixation of the femur places the femoral vessels and the femoral, sciatic, and saphenous nerves at risk. The femoral artery anatomy has been thoroughly studied and safe zones for femoral external fixator pin placement have been established [10,11]. Vascular and neurologic injuries are potentially devastating iatrogenic complications. Provisional fracture reduction and restoration of limb alignment will help reestablish normal anatomic landmarks and neurovascular anatomy prior to external fixator placement.

## Case report

**Figure 1:**
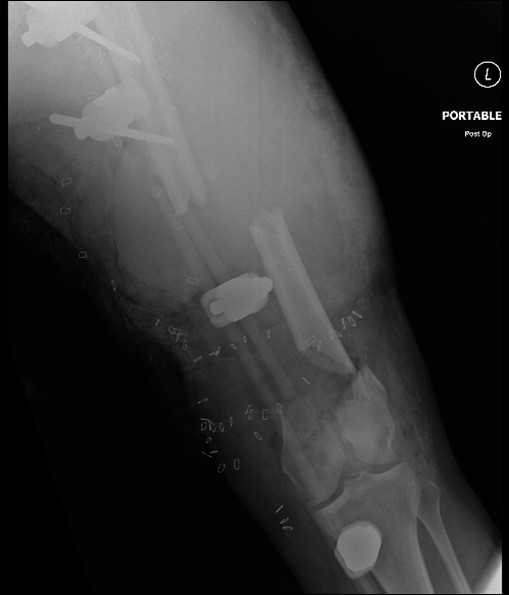
**Type IIIB open segmental femur fracture with ex-fix in place**.

**Figure 2:**
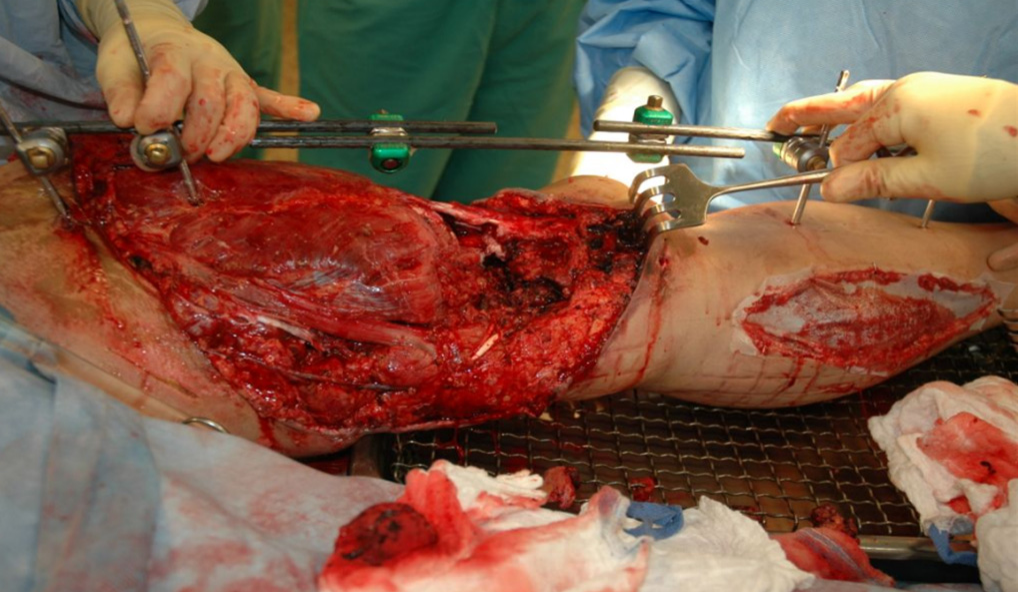
**Severe associated soft tissue injury**. Vascular compromise is due to the distal femoral fixator pin within the zone of injury.

The patient is a 21-year-old female involved in a high speed, helmeted motorcycle accident. After initial evaluation at a community hospital she was transferred emergently to a Level 1 care center. On arrival she was evaluated by the orthopedic and trauma surgery teams, and noted to have a severely deformed left lower extremity with a significant soft tissue injury and active proximal bleeding. She was taken to the operating room where bleeding was isolated to small branches of the femoral artery at the level of an inguinal crease laceration. Hemostasis was obtained by the trauma surgery team. Concurrently, the orthopedic surgery team placed a spanning external fixator across her comminuted, Type IIIB open femur fracture. Proximal fixation was within the proximal femur and distal fixation in the tibia, spanning the knee joint (Figure 1). Of the pins within the femur, the proximal pin was placed using standard percutaneous technique and the distal pin placed under direct visualization through the large soft tissue defect (Figure 2). Prophylactic four compartment leg fasciotomies were performed as well.

**Figure 3:**
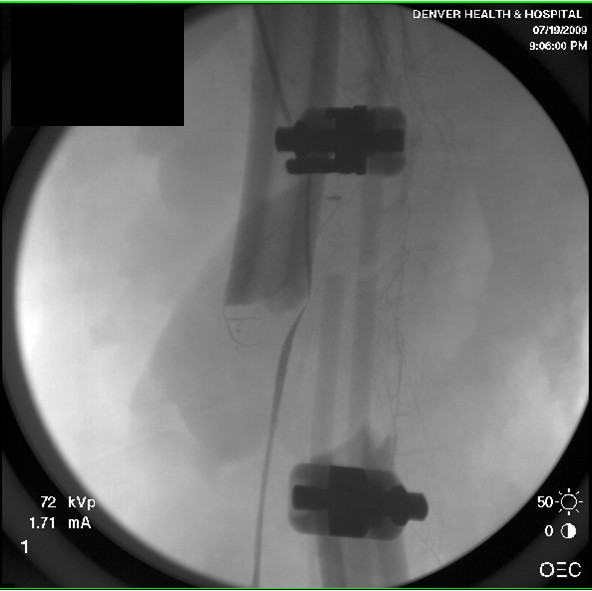
**Intraoperative angiogram showing the SFA lying lateral to the distal femoral fixator pin with diminished distal flow**.

At the time of her index procedure distal pulses were absent and an on-table angiogram was performed. Initially, this showed limited flow below the level of the trifurcation, which improved with application of local vasodilatory agents. Over the preceding 12 hours her dorsalis pedis pulse was lost and a CT angiogram performed. This study suggested the SFA was lying lateral to the distal of the two femoral ex-fix pins, becoming kinked as the anatomic course of the vessel is medial to the femur. The altered path of the SFA was also recognized on retrospective review of the initial angiogram (Figure 3). We emergently returned to the operating room and confirmed the findings of the CT angiogram, with a palpable SFA pulse noted to the level of the pin then no distal pulse. The pin was removed after retraction of the vessel, then replaced through the same location in the bone allowing the soft tissues and SFA to lie medial to the pin. This relieved the kinking of the artery and provided palpable distal flow.

This patient had a protracted hospital course in treatment of her severe left thigh injury. She underwent femoral fixation with a locking lateral femur plate, which required later revision by shortening the plate and removing the devitalized intercalary segment. She eventually underwent split thickness skin grafting of the wound. No further vascular complications were noted during her care.

## Discussion

We report a case of iatrogenic superficial femoral artery occlusion after placement of a knee-spanning external fixation frame in a Type IIIB open femur fracture with significant soft tissue degloving. Despite proper pin insertion technique under direct visualization, a resulting vascular compromise was diagnosed and this necessitated pin removal and reinsertion with superficial femoral artery repositioning to restore proper flow. As seen in this case, anatomic consideration relies heavily on normal and expected courses of vessels and nerves, and in the setting of severe trauma these relationships are disrupted and may not be reliable.

Rates of vascular injury after skeletal external fixation are unknown; however, there are multiple case reports in the literature highlighting this potentially devastating complication. Vessel injury has been described secondary to direct or partial laceration, indirect compression and impingement, and erosion [12-17]. Paul et al. reported four iatrogenic vascular injuries after external fixation frame placement in 121 lower extremity fractures for an incidence of 3.3% [12]. Dhal et al. reported on thirteen pseudoaneurysms associated with extremity trauma, of these five (38.5%) were caused by external fixation pins [13]. Signs of vascular injury following external fixator pin insertion included bleeding from insertion site, loss of distal pulse, a pulsatile mass, or ischemia. In addition, the diagnosis may be delayed secondary to collateral flow or the injury may not occur until weeks or months after insertion as seen in erosive injuries [12-14,16].

Recognition of vascular injury following surgical stabilization of extremity trauma is critical for limb viability and failure to diagnose may lead to devastating complications. This case highlights the importance of both pre- and postoperative vascular exams following procedures that risk vascular injury, especially in the critically injured where an exam may be unreliable. The clinical presentation of a vascular injury may be occult and a high index of suspicion must remain even in the setting of palpable pulses [12,18]. Hard signs of vascular injury include pulselessness, pallor, paresthesias, palpable or audible bruit, and expanding hematoma, however these are not always present in a vascular injury. Any change in vascular status should prompt a thorough investigation with Ankle-Brachial Indices (ABI), duplex ultrasonography, CT angiography, and/or arteriography. Early vascular surgery consultation is also recommended. Management may range from observation to open or endovascular reconstruction depending on injury and perfusion. Surgical revascularization is needed within 6 hours of ischemic injury to avoid permanent soft tissue damage [18].

## Conclusion

This case highlights the dangers associated with damage control orthopedics, especially when severe trauma alters normal local anatomy. Careful assessment of external fixator pin placement is crucial to avoiding iatrogenic injury. We recommend a thorough vascular examination pre-operatively and prior to leaving the operating room, which allows any abnormalities to be further evaluated while the patient remains in a controlled environment. When an unrecognized iatrogenic injury occurs, serial postoperative neurovascular examinations allow early recognition and corrective actions.

## Consent

Written informed consent was obtained from the patient for publication of this Case report and any accompanying images. A copy of the written consent is available for review by the Editor-in-Chief of this journal.

## Competing interests

The authors declare that they have no competing interests. No external sources of funding were provided in preparation of this manuscript. The views expressed in this article are those of the authors and do not reflect the official policy or position of the Department of the Navy, the Department of Defense, or the United States Government.

## Authors' contributions

GS completed the literature review and composed the background portion of the manuscript. MF authored the case, provided manuscript compilation, and was responsible for producing the attached figures. SM provided the concept and design of this case report, critical review of the manuscript, and gave final authorization for submission of the manuscript in current form. All authors have read and approved the final manuscript.
